# The Effects of a Web-Based Need-Supportive Intervention for Physical Education Teachers on Students’ Physical Activity and Related Outcomes: A Randomized Controlled Trial

**DOI:** 10.3390/children12010056

**Published:** 2025-01-03

**Authors:** Hasso Paap, Andre Koka, Pille-Riin Meerits, Henri Tilga

**Affiliations:** Institute of Sport Sciences and Physiotherapy, Faculty of Medicine, University of Tartu, 51008 Tartu, Estonia; andre.koka@ut.ee (A.K.); pille-riin.meerits@ut.ee (P.-R.M.); henri.tilga@ut.ee (H.T.)

**Keywords:** need-support, psychological needs, motivation, intervention, adolescents, self-determination theory, physical education

## Abstract

Background/Objectives: Globally, adolescents are insufficiently active, highlighting the need for effective strategies to enhance their activity levels. This study evaluated a web-based intervention program designed for physical education (PE) teachers, aimed at fostering students’ basic psychological needs—autonomy, competence, and relatedness—in PE settings. Methods: Secondary school PE teachers and their students were randomly assigned into either an experimental (teachers *n* = 36; students *n* = 463, Mage = 13.94, SD = 0.88) or a control group (teachers *n* = 49; students *n* = 820, Mage = 13.93, SD = 0.87). Teachers in the experimental group underwent a 4-week web-based intervention program focused on autonomy-, competence-, and relatedness-supportive motivational techniques. Students completed questionnaires assessing psychological measures and self-reported physical activity (PA) before and after the teachers’ training. Results: Results indicated that students in the experimental group perceived significantly higher autonomy, competence, and relatedness support compared to their control group counterparts at post-test. Conclusions: Consequently, the web-based need-supportive intervention was effective in promoting need-supportive teacher behavior. As the developed training for PE teachers increased students’ perceptions of need support from their PE teachers, it holds promise for future PE teacher education. Furthermore, the web-based format offers scalability for broader implementation.

## 1. Introduction

According to World Health Organization (WHO) guidelines, it is recommended that children and adolescents participate in a minimum of 60 min of moderate- to vigorous-intensity PA each day [1]. However, research by Guthold and colleagues [2] revealed that merely 19% of the world’s 11–17-year-olds were sufficiently physically active. This alarming statistic underscores the argument that the PA of children and adolescents should be a cross-disciplinary priority [3].

Physical education (PE) is an educational discipline focused on nurturing an individual’s physical, cognitive, and psychosocial skills. A robust PE program provides children and adolescents with regular physical activity (PA), knowledge, skills, and motivation, fostering a sustained commitment to diverse activities as an integral component of a health-conscious lifestyle [4].

Notably, early positive encounters within PE classes play a pivotal role in shaping a child’s supportive attitude toward PA, serving as the foundation for future engagement [5,6]. Given the low levels of PA among children and adolescents, PE classes represent a critical opportunity to effect change, fostering positive attitudes toward PA that can carry over into adulthood and benefit society as a whole.

The self-determination theory (SDT) posits that fulfilling an individual’s three basic psychological needs (BPNs) contributes significantly to intrinsic motivation and overall well-being [7]. These needs include autonomy (the desire to exert control over events in their life); competence (the desire for recognition of capabilities and skill development); and relatedness (the desire for emotional connections with others). When these needs are fulfilled, they positively impact intrinsic motivation and mental well-being; conversely, unmet needs lead to reduced motivation and overall well-being [7]. A meta-analysis by Howard and colleagues within the school context [8] reveals that intrinsic motivation is associated with student success and well-being, whereas identified regulation is closely linked to persistence. Introjected regulation relates to persistence and performance but also correlates with negative well-being indicators. External regulation is associated with lower well-being, and amotivation is linked to adverse outcomes. Therefore, identified regulation and intrinsic motivation are essential for effective school adjustment [8]. Ryan and colleagues [9] found that the satisfaction of BPN establishes a strong foundation for the emergence of intrinsic motivation toward PA in PE classes. When children and adolescents experience fulfillment of their needs for autonomy, competence, and relatedness in PE, they are more likely to engage in PA, thereby supporting a sustainable and health-conscious lifestyle.

PE teachers, according to White and colleagues [6], impact students’ intrinsic motivation by addressing their BPNs. A supportive learning environment, characterized by student autonomy, choices, recognition, and clear explanations regarding the importance of physical exercise, significantly contributes to the satisfaction of BPNs. Conversely, controlling behavior from teachers leads to the frustration of BPNs and student dissatisfaction [6].

Promoting autonomy, competence, and relatedness is crucial for enhancing students’ intrinsic motivation and increasing their participation in PE [10]. When students perceive themselves as physically capable, receive support, and actively participate in decision-making, they are more likely to enjoy participating in PE classes [11]. Decreasing external control leads to improved intrinsic motivation, overall well-being, performance, and self-esteem [7]. Teachers should adopt an individualized approach, considering student abilities and preferences, while aligning with lesson objectives [7]. Teachers’ controlling behavior is negatively correlated with intrinsic motivation and identified regulation, but positively correlated with introjected and external regulation, as well as amotivation [12,13]. Supporting autonomy within PE classes improves both classroom dynamics and student performance [14].

Research grounded in SDT has consistently demonstrated that students who excel in PE are more intrinsically motivated. Furthermore, motivated students are more willing to engage in leisure time PA (LTPA) [15,16]. The trans-contextual model of motivation (TCM) provides a comprehensive framework for understanding how motivational transfer occurs across diverse contexts. Specifically, it explains how intrinsic motivation experienced within a specific context (e.g., motivation in PE) transfers to motivation in related contexts outside of the school environment (e.g., motivation for LTPA). Positive experiences in PE foster overall interest in PA [17].

Autonomy support in PE classes is essential. However, this does not imply a lack of discipline or teacher withdrawal [18]. Numerous studies have highlighted the positive impact of training teachers in autonomy-supportive behaviors. In a school-based intervention, adolescents were empowered to be physically active both within and outside of school [19]. The intervention emphasized need-supportive teaching principles and resulted in significant improvements in sociocultural determinants and motivational outcomes related to both PE and LTPA. Tilga and colleagues found that a web-based autonomy-supportive intervention significantly improves PE teachers’ self-reported autonomy-supportive behavior and teaching efficacy, which subsequently enhances students’ psychological need satisfaction and intrinsic motivation [20]. Similarly, Zimmermann and colleagues found that cognitive autonomy support was a positive predictor of students’ self-efficacy and intrinsic value [21]. Another intervention assessed the impact of autonomy support on students’ PA outcomes, showing positive effects both during PE classes and beyond school hours [22]. Importantly, these studies underscore that PE teachers can also support students’ LTPA [19,22].

Online need-supportive interventions provide opportunities for greater personalization and self-paced learning [23]. Evidence from intervention studies has demonstrated the effectiveness and positive impact of online approaches for PE teachers’ outcomes [20], as well as for student outcomes [24]. Notably, a letter to the editor discusses enduring effects observed over a longer period.

This study aimed to assess the effectiveness of an online IP based on the classification system of motivational behaviors [25] for PE teachers, specifically evaluating significant differences in variables such as perceived autonomy, competence, and relatedness support, as well as controlling behavior from teachers. The hypothesis was that, at post-test, students in the experimental group would perceive greater need support from their teachers, higher satisfaction of BPNs, increased autonomous motivation toward PA in PE and leisure time and greater self-reported PA. Conversely, they would perceive less controlling behavior from teachers and less frustration of BPNs compared to students in the control group. Key study variables included students’ perceptions of BPN satisfaction and frustration, autonomous motivation in PE, motivation in leisure time, and self-reported PA. Should the intervention prove effective in modifying teacher behaviors, it could be seamlessly integrated into PE teacher training programs. To the best of our knowledge, while previous studies have examined support for autonomy, this study distinctly addresses all three BPNs of autonomy, competence, and relatedness.

## 2. Materials and Methods

### 2.1. Participants and Design

**Figure 1:**
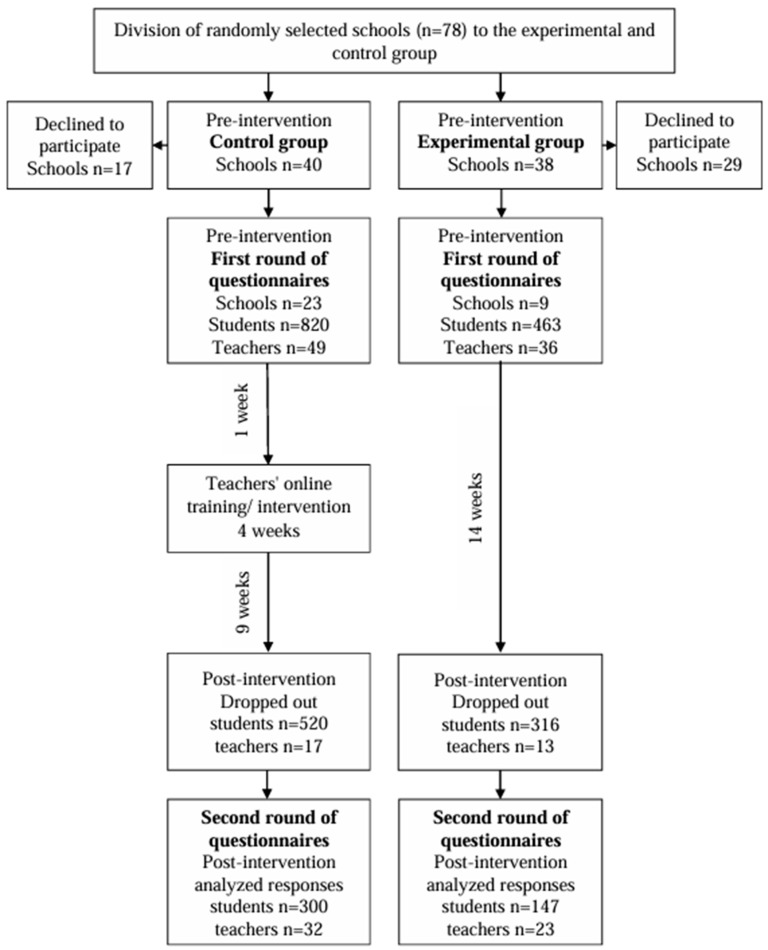
Participant flow diagram and overall study design.

The study adopted a cluster-randomized controlled design with two study groups, implementing 1:1 randomization. Participants included secondary school PE teachers and their students, who were assigned to either an experimental group or a control group based on their respective schools (Figure 1). The sample was derived using a systematic random sampling method, utilizing a list of schools available on the State Portal. Secondary schools were randomly selected for both the experimental and control groups, employing a random number generator [26]. The study specifically targeted PE teachers and students at the third level of secondary school (7th–9th grade), with eligibility criteria allowing for student participation in PE classes without restrictions.

The study comprised 38 schools in the experimental group and 40 schools in the control group (Figure 1). Of the 85 teachers who participated in the study, 49 taught control group students, while 36 taught experimental group students. PE teachers in the experimental group engaged in a 4-week online IP. Students were blinded to allocation. The study involved 1283 students (M_age_ = 13.93). Among the participants, 553 were boys (M_age_ = 13.94, SD = 0.88), 725 were girls (M_age_ = 13.93, SD = 0.87), and 5 students did not indicate their gender (M_age_ = 14.60, SD = N/A). Sample size estimation using G*Power Version 3.1.9.7 [27] indicated that 269 participants were needed for ANCOVA testing to achieve 80% power for detecting medium effects, at a significance level of α = 0.05, considering one covariate. The initial sample size exceeded the minimum requirement due to potential attrition in voluntary online studies (approximately 40%) during subsequent measurements [24].

The students in both study groups simultaneously completed a questionnaire before and after the intervention. The questionnaire was administered online, with the link distributed via each participating school’s principal or head of studies. The survey was conducted using Google Forms, and it took students an average of approximately 30 min to complete. The study was conducted between September and December 2022.

### 2.2. Ethical Considerations

This research is a part of a broader project (Increasing Physical Activity of School Students through Support of Physical Education Teachers and Parents’ Autonomy; approved by the Research Ethics Committee of the University of Tartu, protocol number: 327/T-4). The CONSORT checklist guided the preparation of this report [28].

Informed consent forms and study information were electronically distributed to each participating school’s principal or head of studies, who subsequently forwarded them to the legal guardians of the students. Participants were informed at the beginning of the online questionnaire that participation is voluntary, and that their anonymity would be guaranteed. To match the responses across two time points, participants were asked for their school’s name, age, grade number, gender, and the first three letters of their mother’s and father’s names. The study did not harm the participants either mentally or physically, and invasive research methods were not used.

### 2.3. The Structure of the IP

An online training program titled “Teaching Physical Education While Supporting Basic Psychological Needs” was developed for the teachers in the experimental group. Conducted over a 4-week period from September to October 2022, the training program took place on the Moodle platform. Participation was voluntary and free of charge, with no financial incentives provided to participants.

The primary objective of this training program was to provide PE teachers with knowledge and skills to effectively support their students’ BPNs for autonomy, competence, and relatedness, with the aim of enhancing students’ autonomous motivation toward PA.

The training program consisted of weekly instructional videos, totaling 21 distinct behavioral techniques (Supplementary Materials). Each video, lasting approximately 4 to 5 min, followed a structured format. Firstly, an introduction to the psychological need addressed by the technique was provided; secondly, a brief overview of the technique was presented; thirdly, a demonstration of how the technique could be misapplied was shown; and finally, a demonstration of the correct and effective use of the behavior technique was presented. The content of the educational videos was based on the motivational techniques described by Teixeira and colleagues [25]. Example videos can be accessed upon request by contacting the authors.

Experimental group PE teachers actively participated in the course, consistently applying the behavioral techniques introduced during their weekly classes. Teachers were asked to implement the techniques shown in the training videos on a weekly basis throughout the intervention. The fidelity of the intervention was evaluated through three distinct approaches. Firstly, students’ perceptions of their PE teachers’ autonomy-supportive and controlling behaviors were assessed both before and after the intervention. Secondly, PE teachers in the experimental group were asked to write weekly forum posts detailing their implementation of autonomy-supportive practices during a four-week intervention period. Thirdly, the teachers completed short knowledge-check tests.

### 2.4. Measures

In this quantitative study, various psychological variables in two measuring occasions were assessed using 7-point Likert scales (except for questions related to PA, which used a 6-point scale). On the 7-point scale, a response of “1” indicates complete disagreement with the statement, while “7” indicates complete agreement with the statement. The reliability of the scales in the questionnaires was assessed by Cronbach’s alpha coefficient [29].

Perceived need support. Perceived autonomy support from PE teachers was assessed using a version of the shortened Perceived Autonomy Support Scale for Exercise Setting (PASSES) questionnaire [30]. This measure has been adapted into the Estonian language and used on adolescents [31]. The subscale consisted of 7 items (e.g., “I feel that my PE teacher provides me with choices, options, and suggestions about whether to do PA”). Previous studies have shown that the scale evaluating students’ perceived autonomy support from teachers is valid and reliable [31]. The Cronbach’s alpha for the subscale was above 0.90, indicating very good internal consistency.

Perceived competence and relatedness support were measured by the Perceived Psychological Needs Support questionnaire from the Teacher in Physical Education [10] that has been adapted into the Estonian language and used on adolescents [32]. The subscale for competence support consisted of 4 items (e.g., “I feel that my PE teacher helps me to improve in PA”) and the subscale for relatedness support consisted of 5 items (e.g., “I feel that my PE teacher supports me”). Previous studies have demonstrated that this measure is reliable and valid [32,33]. The Cronbach’s alphas for these subscales were above 0.90, indicating very good internal consistency.

In these subscales, a higher score means a higher perceived need support.

Perceived controlling behavior. Perceived controlling behavior from PE teachers was assessed using the multidimensional controlling coach behaviors scale [34] that was adapted to PE and the Estonian language [35]. The subscale consisted of 5 items (e.g., “My teacher uses the threat of punishment to keep me in line during lesson”). This measure has been shown to be reliable and valid, and has it been used on adolescents [36]. The Cronbach’s alpha for this subscale was above 0.80, indicating good internal consistency. In this subscale, a higher score means a higher perceived controlling teaching behavior.

BPN satisfaction and frustration. Students’ BPN satisfaction and frustration were assessed using the Basic Psychological Need Satisfaction and Need Frustration Scale [37], adapted to the context of PE [38]. In total, 24 statements were used in this subscale. All items were preceded by a common stem “When I engage in PA in PE classes…”. Example items are: “…I feel that my decisions reflect what I really want” (autonomy satisfaction); “…I feel forced to do many things I wouldn’t choose to do” (autonomy frustration); “…I feel I can successfully complete difficult tasks” (competence satisfaction); “…I feel insecure about my abilities” (competence frustration); “…I experience a warm feeling with the people I spend time with” (relatedness satisfaction); “…I feel the relationships I have are just superficial” (relatedness frustration). Previous studies have shown that the scale is valid and reliable [36,39]. The scale has been used in the Estonian language and with adolescents [36]. The Cronbach’s alpha for the need frustration subscale was above 0.8 and for the need satisfaction subscale above 0.90, indicating good to very good internal consistency. In these subscales, a higher score means higher perceived need satisfaction or need frustration, depending on the item.

Students’ autonomous motivation in PE and leisure time. Students’ autonomous motivation in PE was assessed using the Perceived Locus of Causality questionnaire [40]. Autonomous motivation toward PA in leisure time was assessed by the Perceived Locus of Causality (PLOC) questionnaire [41]. In this subscale, there were 4 statements related to PE and 4 statements related to leisure time. Only statements assessing intrinsic motivation and identified regulation, which constituted autonomous motivation, were used in the study. All items had a common stem “I participate in PE class/I am physically active in my free time…” and were followed by statements assessing intrinsic motivation (“…because I enjoy it”) or identified regulation (“…because it is important to me”). Previous studies have shown that the scale measuring students’ autonomous motivation in PE and leisure time is valid and reliable [39,42]. The scale has been used in the Estonian language and with adolescents [42]. The Cronbach’s alpha for the autonomous motivation in the PE subscale was above 0.8 and for the autonomous motivation in the leisure time subscale above 0.90, indicating good to very good internal consistency. In these subscales, a higher score means higher perceived autonomous motivation in either PE or leisure time, depending on the item.

Students’ PA. Students’ PA was assessed using the Leisure Time Exercise Questionnaire [43], comprising 2 questions. These questions are as follows: “How often did you engage in sports and/or vigorous PA during your leisure time for at least 20 min continuously over the past 5 weeks?” and “What was the frequency of your engagement in sports and/or vigorous PAs during your leisure time for at least 20 min continuously over the past 5 weeks?”. Students responded on a 6-point scale ranging from 1 (“Never”) to 6 (“Every day”). A higher score indicates a greater level of PA. Previous studies have shown that the scale measuring students’ PA is valid and reliable, and it has been used with Estonian adolescents [44]. The Cronbach’s alpha for this subscale was above 0.80, indicating good internal consistency.

### 2.5. Data Analysis

The data analysis was performed using JASP (version 0.17.1; University of Amsterdam, Amsterdam, the Netherlands). Before conducting the data analysis, the normal distribution of the data was checked, with the skewness and kurtosis values expected to fall within the range of −2 to 2 [45]. For all the variables used in the study, the mean values (M) and standard deviations (SD) were calculated. The chi-square test was used to examine differences in participants’ gender and grade level across study groups. The internal reliability of the questionnaires’ subscales was assessed by Cronbach’s alpha values [46].

Two types of *t*-tests were used to evaluate mean differences in the variables: paired samples *t*-test and independent samples *t*-test. The paired samples *t*-test was used to assess within-group changes in the study variables before and after the intervention. The independent samples *t*-test was used to assess pre-intervention differences in the study variables between the experimental and control groups and to evaluate differences between participants based on attrition.

Analysis of covariance (ANCOVA) was utilized in the data analysis, with the pre-intervention scores used as a covariate in each analysis. ANCOVA was used to identify differences between the experimental and control groups in the study variables after the intervention.

## 3. Results

### 3.1. Preliminary Analysis

Descriptive statistics. Skewness coefficients ranged from −0.894 to 0.527, and kurtosis coefficients ranged from −0.770 to 0.508. These findings suggest that all investigated variables adhere to a normal distribution [45]. To assess internal reliability, Cronbach’s alpha was employed for the questionnaire scale, yielding values between 0.75 and 0.94. These results affirm that the evaluated scales demonstrate satisfactory to very high levels of reliability in measuring outcomes [46]. The ranges of the skewness and kurtosis coefficients and the values of internal reliability span both pre- and post-intervention measurements.

Comparisons of the baseline characteristics between the study groups. Before the intervention, autonomous motivation towards LTPA (*t* = 3.41, *p* = 0.001) and self-reported PA (*t* = 2.16, *p* = 0.031) were higher in the experimental group than in the control group. No other statistically significant differences were observed between the control and experimental groups at baseline (*p* = 0.068–0.995).

Characteristics between the participants who remained in the study and those who were lost in post-test. Pre-intervention differences in study variables between participants who completed the study and those who dropped out were also examined. There were no statistically significant differences in the mean values of any of the variables examined (*p* = 0.074–0.460).

Intragroup changes in study variables before and after the intervention. Table 1 displays the intragroup changes in study variables between the experimental and control groups before and after the intervention. Following the intervention, the control group reported significantly lower levels of perceived autonomy (*t* = 3.31, *p* = 0.001), competence (*t* = 4.48, *p* < 0.001), and relatedness support (*t* = 2.59, *p* = 0.010) in PE class compared to the pre-intervention period.

### 3.2. Main Analysis

Differences in psychological variables between experimental and control groups at post-test. The results of the covariate analysis (ANCOVA) (Table 2) indicated that students in the experimental group perceived significantly higher levels of autonomy support (*F*(1, 444) = 4.09, *p* = 0.044), competence support (*F*(1, 444) = 7.27, *p* = 0.007), and relatedness support (*F*(1, 444) = 4.10, *p* = 0.044) from their PE teachers after the intervention, compared to students in the control group. No statistically significant differences were found between the two groups in the other study variables (*p* > 0.05).

## 4. Discussion

The aim of this study was to investigate whether the implementation of an IP for PE teachers leads to significant differences in PA-related outcomes between students in the experimental group and those in the control group. Our findings provide novel evidence of the efficacy of the developed IP in enhancing students’ perceptions of need-support from their PE teachers, a dimension that has been underexplored in previous research. This online program not only demonstrates its effectiveness but also holds promise for integration into PE teacher training, as the web-based format offers scalability for broader implementation, ultimately enhancing PE teachers’ need-supportive behavior toward their students.

Before testing the study hypotheses, we assessed randomization success in assigning students to study groups. Specifically, we examined whether there were significant differences in the measured variables between the students in the experimental group and those in the control group prior to the intervention. The experimental group students showed higher autonomous motivation for LTPA and self-reported PA than the control group students. These initial motivation differences likely influenced student perception of teacher behavior after the intervention. For example, students who generally display greater motivation for physical activities may perceive their PE teacher’s behavior more favorably. Several studies have demonstrated such reciprocal effects between student motivation and the perception of teacher behavior [47,48,49]. Furthermore, elevated levels of autonomous motivation toward LTPA have been associated with increased PA levels among students [50]. Importantly, other measured variables were similar between groups before the intervention, ensuring comparability at the study’s outset. Other comparable studies have yielded no statistically significant differences between study groups before the intervention [19,36].

In the context of attrition assessment, we compared the measured variables at the study’s outset between students who withdrew and those who completed the study. Prior to the intervention, no statistically significant differences were observed in the mean values of these variables. Thus, it can be asserted that the dropout rate was not associated with significantly different perceptions of participants’ psychological experiences or significantly different levels of PA. Our conclusion is that students who remained in the study exhibited similar psychological experiences compared to those who dropped out. A comparable intervention study conducted by Tilga and colleagues [20] also reported no significant differences in measured variables before the intervention between the students who dropped out and those who completed the study.

To test our hypothesis, the initial task was to identify within-group changes in measured variables among students in both the experimental and control groups before and after the intervention. Notably, the control group perceived significantly lower levels of autonomy support, competence support, and relatedness support in PE classes after the intervention, while the experimental group showed no significant changes. This suggests that the intervention may have helped to maintain the baseline levels of perceived need-support for the experimental group, as the control group demonstrated decreased levels of these variables. Interestingly, our findings differ from a similar study by Aelterman and colleagues [51], which not only focused on autonomy support but also monitored changes in the use of structure, with the measured use of structure being comparable to competence support. Overall, after the intervention, there was a significant improvement in the experimental group’s students’ perceptions of both autonomy and competence support.

The subsequent task to test our hypothesis was comparing the measured variables between the experimental group and the control group after the intervention, while considering pre-intervention indicators. Post-intervention assessments revealed that students in the experimental group perceived significantly higher levels of autonomy support, competence support, and relatedness support in PE classes compared to their counterparts in the control group. Previous similar IPs in PE have also consistently demonstrated students’ increased perception of autonomy support, a critical factor for promoting PA both during PE classes and leisure time [22,52]. Supporting autonomy, competence, and relatedness needs fosters intrinsic motivation among students, leading to greater engagement in PE [10]. When students perceive higher levels of support for their BPNs, it is highly probable that they enjoy participating in PE classes more, ultimately contributing to increased PA levels in PE [11] and out-of-school PA [16,17]. Our study’s findings align with previous analogous research, affirming the effectiveness of the intervention in enhancing students’ perception of teacher support for their BPNs.

Knittle and colleagues [53] highlighted the effectiveness of motivation-supportive IPs in enhancing both psychological experiences and PA. Meerits and colleagues [33] designed an online need-supportive IP for parents which did not significantly impact PA, but increased intrinsic motivation and increased and decreased controlled forms of motivation in the experimental group. The results from the current study align with the results from Meerits and colleagues, as we also found no significant increase in the PA levels among students in the experimental group. However, it is noteworthy that students in the experimental group reported higher perceptions of autonomy, relatedness, and competence compared to their counterparts in the control group. This confirms earlier research results [53] emphasizing the utility of motivation-supportive IPs in fostering positive psychological experiences.

While evaluating the effectiveness of our intervention, we found no significant increase in PA among students in the experimental group compared to the control group nor any significant changes in motivation for PE and LTPA within the experimental group. However, the intervention may have effectively maintained baseline motivation levels in the experimental group, which is crucial for fostering long-term engagement in PA. It is important to note that the duration of our study might have been insufficient to detect substantial changes. Establishing a perception of support for BPNs likely requires more time, which could influence subsequent psychological experiences. Additionally, seasonal variations may have affected students’ PA levels; specifically, weather conditions at the study’s outset in autumn were more favorable for outdoor PA, whereas the winter period toward the study’s end might have posed challenges for maintaining consistent PA levels. Therefore, the ability to sustain baseline motivation levels among students can be viewed as a positive indicator.

### Strengths, Limitations, and Future Directions

The strengths of this study include the large number of students participating and the comprehensive and concise study variables (eight different psychological traits examined). By involving students from randomly selected schools across Estonia, the study’s findings can be broadly applied to understand the relationships between psychological experiences and PA within this age group. Furthermore, the study’s strength lies in its theoretical foundation, specifically the behavioral techniques used during the online training of the experimental group teachers [25]. Additionally, this study benefits from its online approach, which allowed the inclusion of a larger number of participants (both students and teachers) compared to an exclusively in-person study design. The web-based format offers scalability for broader implementation and is also cost-effective, supporting sustainable interventions with minimal material costs while reaching a substantial number of participants.

A limitation of this study is the high dropout rate among students who initially participated but subsequently withdrew after the first round of questionnaire responses. To improve future research, we recommend using a more personalized contact method for questionnaire delivery to participants. Additionally, the findings from Tilga and colleagues’ [36] study highlight the effectiveness of autonomy-supportive interventions, particularly those that utilize a blended learning approach combining face-to-face and online instruction. This hybrid model has been shown to enhance students’ intrinsic motivation while reducing the impact of extrinsic motivators. Drawing on this knowledge and our experiences in the study, we propose a blended intervention framework for PE in future research. A hybrid approach that integrates online intervention with in-person sessions may improve the effectiveness of the intervention [36] and therefore, is recommended for similar studies moving forward.

It is essential to acknowledge that the evaluation of intervention effectiveness relied solely on self-reported measures from participating students. Consequently, this approach did not provide a comprehensive overview of the extent to which teachers successfully implemented the behavior techniques acquired during online training within the classroom setting. As we did not plan for classroom observations in this study, we are unable to assess the degree to which teachers applied these techniques. In future similar studies, it is recommended to observe teachers’ behavior both before and after the intervention, as advocated by Barkoukis and colleagues [22] based on their own research.

Furthermore, the online nature of our study introduces another constraint: the absence of direct contact between researchers and participating students and teachers. This lack of real-time interaction may have contributed to the ease with which participants dropped out during the study.

In this study, autonomy support was measured as a unidimensional construct. Future studies would do well by measuring autonomy support as a multidimensional construct [21,54,55]. The reason for this is that a multidimensional approach to autonomy support might enable us to describe larger amount of variance in basic psychological needs satisfaction.

In further intervention studies, it is advisable to include an additional measurement occasion to capture the potential long-term effects of the intervention on PA behavior. For example, Tilga and colleagues demonstrated that their autonomy-supportive intervention for PE teachers had long-term effects on students’ perceptions of autonomy-supportive teacher behavior, perceived autonomy support, and intrinsic motivation towards PE classes [24].

For future investigations, we suggest considering a more nuanced list of behavior techniques classified by Ahmadi and colleagues [56]. It specifically addresses the support and hindrance of BPNs within an educational context. This alternative framework could offer valuable insights beyond the health-oriented techniques previously outlined by Teixeira and colleagues [25]. Additionally, future research should explore the benefits of need-supportive intervention programs for teachers, as previous studies have indicated that teachers also gain advantages from need-supportive teaching [20,57].

## 5. Conclusions

This study provides evidence of the efficacy of the developed intervention program for PE teachers in enhancing students’ perceptions of need-support from their PE teachers. Post-intervention, the control group students experienced reduced perceptions of autonomy, competence, and relatedness support, whereas the experimental group showed no such changes. After the intervention period, students in the experimental group reported increased levels of autonomy, competence, and relatedness support within the PE class, as compared to their counterparts in the control group. Therefore, this online program holds promise for integration into PE teacher training, as the web-based format offers scalability for broader implementation, ultimately enhancing PE teachers’ need-supportive behavior toward their students.

## Figures and Tables

**Table 1:** Intragroup changes in study variables before and after the intervention (control group *n* = 300, experimental group *n* = 147).

Variable/Group	Pre-Intervention M (SD)	Post-Intervention M (SD)	*t*	*p*
Autonomy support				
Control group	5.15 (1.34)	4.92 (1.32)	3.31	0.001
Experimental group	5.23 (1.33)	5.26 (1.22)	1.19	0.238
Competence support				
Control group	5.31 (1.40)	5.01 (1.39)	4.48	<0.001
Experimental group	5.34 (1.42)	5.38 (1.32)	0.72	0.472
Relatedness support				
Control group	5.09 (1.40)	4.98 (1.36)	2.59	0.010
Experimental group	5.18 (1.42)	5.29 (1.23)	0.40	0.688
Controlling behavior				
Control group	4.49 (1.18)	4.36 (1.23)	1.40	0.162
Experimental group	4.49 (1.14)	4.28 (1.18)	1.26	0.211
Satisfaction of BPN				
Control group	4.78 (1.21)	4.74 (1.16)	1.07	0.284
Experimental group	4.85 (1.21)	4.88 (1.21)	1.62	0.108
Frustration of BPN				
Control group	3.41 (1.08)	3.41 (1.07)	−0.83	0.405
Experimental group	3.49 (1.18)	3.46 (1.24)	−0.70	0.483
Autonomous motivation in PE				
Control group	4.83 (1.59)	4.74 (1.49)	1.29	0.198
Experimental group	5.00 (1.58)	5.18 (1.50)	0.93	0.355
Autonomous motivation in LT				
Control group	5.07 (1.52)	5.02 (1.49)	−0.44	0.657
Experimental group	5.36 (1.37)	5.23 (1.50)	1.29	0.199
Physical activity				
Control group	3.89 (1.26)	3.90 (1.15)	−1.10	0.273
Experimental group	4.05 (1.23)	3.96 (1.30)	−0.19	0.852

Notes. PE = physical education; LT = leisure time; BPN = basic psychological needs.

**Table 2:** Differences in psychological variables between the experimental and control group at post-test.

Variable	Control Group (*n* = 300) M (SD)	Experimental Group (*n* = 147) M (SD)	*F*(1, 444)	Partial η^2^	*p*
Autonomy support	4.92 (1.32)	5.26 (1.22)	4.09	0.009	0.044
Competence support	5.01 (1.39)	5.38 (1.32)	7.27	0.016	0.007
Relatedness support	4.98 (1.36)	5.29 (1.23)	4.10	0.009	0.044
Controlling behavior	4.36 (1.23)	4.28 (1.18)	0.13	0.000	0.722
Satisfaction of BPN	4.74 (1.16)	4.88 (1.21)	0.02	0.000	0.904
Frustration of BPN	3.41 (1.07)	3.46 (1.26)	0.11	0.000	0.740
Aut. motivation PE	4.74 (1.49)	5.18 (1.50)	1.99	0.005	0.159
Aut. motivation LT	5.02 (1.49)	5.23 (1.50)	0.12	0.000	0.730
Physical activity	3.90 (1.15)	3.96 (1.30)	0.00	0.000	0.981

Notes. PE = physical education; LT = leisure time; BPN = basic psychological needs; Aut. Motivation = autonomous motivation; Partial η^2^ = partial eta squared, a measure of effect size.

## Data Availability

The datasets generated and analyzed during the current study are available in the Open Science Framework (OSF) repository, https://osf.io/d5ju9/ (accessed on 19 December 2023).

## Supplementary Materials

The following supporting information can be downloaded at: https://www.mdpi.com/article/10.3390/children12010056/s1. Table S1: The description of 21 behavioral techniques used in the intervention for PE teachers.
