# Supplementary material for: The Effects of a Web-Based Need-Supportive Intervention for Physical Education Teachers on Students’ Physical Activity and Related Outcomes: A Randomized Controlled Trial
Source: Children (Basel). 2025 Jan 3;12(1):56. doi: 10.3390/children12010056 (PMC11763398; doi:10.3390/children12010056)
Supplement: Supplementary file 1 [file children-12-00056-s001.zip › children-3309677-supplementary.pdf]

Supplementary Materials

Table S1: 21 behavioral techniques (Teixeira et al., 2020)

	Title	Meaning	Explanation
**I week**	Investigating opinions and perspectives	Questioning the student about the underlying reasons for their behaviour.	Exploring the underlying causes of behaviour contributes to a deeper understanding of behaviour, both from the perspectives of teachers and students.
	Exploring values and goals	Inquiring about the student's important values and long-term interests.	Knowing the long-term interests and values of the student helps to connect current tasks with similar topics, thus engaging the student more effectively.
	Agreeing with understandings and feelings	Empathetic language use in communication with the student.	Show the student that you understand their viewpoints and feelings (joy, fear, concern) and express your own opinion using a positive attitude and language.
	Encouraging asking questions	Encouraging the student to ask questions.	Encouraging asking questions allows for creating an open and collaborative atmosphere, fostering greater trust in communication.
	Explaining expectations	Identifying the student's expectations.	Assisting the student in setting optimal and realistic expectations helps reduce failure and allows the student to perceive themselves as more competent.
	Creating an action plan	Assist the student in developing a specific action plan to achieve desired goals.	Creating an action plan increases the student's self-confidence and reduces failure.
**II week**	Non-controlling and informative language use	Using non-controlling and informative language when communicating with the student.	Informative and non-judgmental, choice-offering language use helps reduce the emergence of guilt and pressure in the student and enhances their willingness to

			cooperate (e.g., using the word 'could' instead of 'should').
	Offering choices	Provide the student with choices from among alternatives that help achieve the same goal.	Offering choices helps the student perceive personal contribution and responsibility.
	Empathetic listening	While listening to the student's opinion, allow them to finish their sentences and, if possible, reflect back the content or emotions expressed.	Empathetic listening enhances trust and demonstrates respect towards the communication partner.
	Optimal challenge	Assist the student in finding a challenge that is realistic, attainable, and meaningful.	Setting an optimal challenge helps prevent excessive failure and reduces the feeling of incompetence.
	Constructive, clear, and relevant feedback	Provide the student with individual, relevant, and non-judgmental feedback (specific and focused on the process of the activity).	The purpose of constructive, clear, and relevant feedback is to encourage the student and inform them how to proceed.
**III week**	Identifying sources of stress for behaviour change	Identifying sources of stress allows understanding how they affect student-related behaviour and goals.	Identifying sources of stress provides both the student and the teacher with the opportunity to understand why certain activities are unappealing to the student and how to collaboratively change such a situation.
	Interest in well-being	Whenever possible, demonstrate an interest in the student's thoughts and understanding, how they are doing, and how significant life events may influence their mood during the lesson.	Showing interest in the student's well-being allows expressing that the student's experiences and opinions are valuable.
	Assistance in seeking social support	Assist the student, if needed, in defining and identifying possible sources (e.g., friends) who could provide them with positive support in pursuing their goals.	Assisting in seeking social support enhances the student's self-confidence in coping with challenges.

	Identifying obstacles	Examine students about what causes obstacles for them. Based on previous experience, explore how obstacles could be overcome.	Identifying obstacles and gradually overcoming them enhances the student's self-confidence and equips them with skills to independently deal with challenges in the future.
	Identifying coping strategies under pressure	Provide students with information on coping with the pressure of criticism and negative feedback and ways to reduce its impact.	The ability to cope with pressure helps prevent a decrease in the student's sense of competence.
**IV week**	Providing a meaningful rationale	Collaboratively finding a meaningful explanation with the student for what needs to be done at the moment.	A rationale that is personalized and explanatory is valuable for the student.
	Encouraging initiative and experimentation	Encourage the student to suggest new activities for experimentation that are optimally challenging.	Encouraging initiative and experimentation promotes learning and skill development and is inherently enjoyable.
	Unconditional care	Express a positive attitude towards the student regardless of whether they succeed or fail in class.	Unconditional caring and offering support fosters the creation of a relaxed atmosphere in communication.
	Continued support	Provide the student with opportunities for ongoing support if needed.	Providing ongoing support and offering specific opportunities helps the student maintain contact with the teacher and creates conditions for success.
	Monitoring progress	Offer the student opportunities for monitoring progress. Collaborate with the student to identify specific ways they can track their own development in performance and receive feedback on it.	Monitoring progress reinforces success and enables the student to be aware of their development.
